# Cleanliness in Dental Practice

**Published:** 1904-01

**Authors:** A. H. Merritt

**Affiliations:** New York.


THE AMERICAN

### Journal of Dental Science.

### CLEANLINESS IN DENTAL PRACTICE.

By DR. A. H. MERRITT, New York.

Read before the New York Alumni Chapter of Psi Omega.

It is a physical impossibility to maintain asepsis at all times in the daily routine of dental practice. Nor is it always necessary. That the occasion demanding it may often arise none will deny, and the intelligent operator is he who recognizes the occasion, and rises to meet it. But cleanliness—cleanliness in its best and broadest sense—is the corner stone of a successful dental practice. A dentist may be skilful, and possessed of an attractive personality, and yet, because of his indifference to this detail never attain to the full stature of a successful practitioner. And there lies the secret of it in that small word detail. It is only by constantly looking after detail that this condition can be maintained. "Eternal Vigilance" is the price of cleanliness, and that is the reason why many fail. Most men feel (and I think dentists are no exception to the rule), that they were born to do great things, forgetting that in reality great things are accomplished only by the closest observance of detail. It is not necessary that one's office be in a fashionable residential neighborhood surrounded by all the evidences of prosperity. This will come with gray hairs and old age, if we have been faithful in that which seems least, but it is necessary that it be in a locality easy of access, that it be comfortably furnished and scrupulously clean. This latter requirement necessitates its being thoroughly renovated at short intervals, and that it be carefully ventilated, dusted and put in order daily. This should be done before the day's work is begun, and again during the lunch hour it should receive a share of attention.

After the patient has left the chair, the instruments that have been in use should be gathered up, taken to the washstand and scrubbed, after which they should be immersed in the solution in which they are to be sterilized and allowed to remain for a few minutes, when they may be removed, carefully dried, and put in their respective places ready for use again. An excellent solution for use in sterilizing instruments and one that is open to few objections, is a 1 per cent. solution of formaldehyde. It is colorless and practically odorless. It should be kept covered when not in use, and changed frequently as it deteriorates when exposed to the air. The glass containing warm water (which should be placed conveniently near as each patient takes the chair), should also be removed, washed and placed in a jar containing a similar solution, and be replaced by one that has been previously treated in the same way. The hot water syringe (that much neglected instrument), should receive the same treatment, as should also the saliva ejector. These will be ready for use with the next patient, and you have the satisfaction of knowing that they are clean and reasonably sterile. This will of course necessitate *keeping* at least two always at hand.

How to keep the burrs clean is a problem which can be solved by always keeping in use two complete sets. Have the set which you have been using removed, mechanically cleaned, disinfected, and ready for use as the next patient takes the chair. Set No. 2 may then be treated in the same manner. To know that your forceps are always sterile at the moment you want to use them, a very satisfactory method is to keep them constantly immersed in a 20 per cent. solution of formalin with borax in excess. This will maintain asepsis, and instruments kept in it will not oxidize. After having been used they should of course be thoroughly cleaned before they are again placed in the solution. In smaller jars kept for the purpose the hypodermic syringe and needles, matrices, nerve broaches and separating files may be kept in this solution where they are always clean and ready for immediate use. This solution should be in glass stoppered jars as it also deteriorates by exposure to the air. Absorbent cotton is also kept most satisfactorily in glass covered jars.

A convenient table for use at the side of the chair, and one which presents a very neat appearance, is an ordinary dining-room side table. This can be obtained in either oak or mahogany to match the office furniture. It should be about a yard long, eighteen inches wide, have one or two large drawers, and a very commodious shelf about two and one-half feet from the floor. This latter makes a most convenient place on which to keep the large glass jars containing the instruments, cotton, etc. The top may be covered with a beveled edged plate glass, on the top of which may be kept the tray in which the instruments that are in daily use are sterilized, and many other things that may be in constant use.

The tseptic dental napkin is indispensable in any office. When a thicker mouth napkin is desired, which I find often is, a very desirable one can be made from bird's-eye linen. This can be bought in rolls of ten yards for about sixty cents, and can be cut in any size desired. For use over the patient a table napkin about two feet square will be found very satisfactory. If there is one thing in which a dentist should be extravagant, it is in his use of clean linen. Let him always have an abundance at hand, and then use it with a prodigality born of that knowledge. An inexpensive and cleanly cover for the bracket table is one made of white blotting paper. This can be cut to conform to the table and as soiled may be turned over and made to do duty for another day. I know there are those who advocate changing the cover with each patient, but I do not think this necessary, except in those cases where it may be advisable to maintain complete asepsis. This has proven in my hands more satisfactory as a cover than napkins, glass or celluloid. Too often if the armamentarium in the dentist's office were examined it would reveal a miscellaneous, ill assorted lot of bottles, of many kinds, and no settled character, some with paper labels, more without any, and all looking as though they might long ago have been retired and placed upon the pension list to advantage. To say the least this condition of things presents a very untidy appearance and one which will not escape the attention of the observing patient. This can be obviated by replacing them with glass stoppered and glass labeled ones of uniform size. These can be ordered in two or three ounce sizes for about three dollars ($3.00) per dozen. Larger sizes at slight additional cost.

Care should always be exercised in one's personal appearance. First impressions are often lasting ones, and one cannot afford to be indifferent in this matter. That "the apparel oft proclaims the man" is all too true, and it becomes especially incumbent upon us as dentists that we avoid even the appearance of untidiness. Personally I am opposed to wearing a white duck coat in the office, even though it may have the red cross upon the sleeves. I have a feeling that it detracts from one's dignity, and lends to the office the atmosphere of the shop. I do not wish to appear dogmatic in this, for I know there are many good men who approve of their use.

The hands and nails must also come in for a large share of attention. The frequency with which they are washed, and their contact with plaster of paris tends to dry the skin, and unless care be used, they present a rough and grimy appearance far from prepossessing. I might add here that they should always be washed in the presence of the patient. Not only is it necessary that they be clean, but it is necessary that the patient should know it. But you say all this is not necessary, that no one is so observing that he will note whether all this care has been taken, or will care if they should.

In reply let me cite two cases that recently came under my observation, and these are not isolated ones. One, a woman, left her dentist because his nails were not clean. The other, also a woman, because she was given a glass of water to rinse her mouth with, on which were still the evidences of its having been used by a previous patient. In both instances these were patients whom any one would be glad to number among his clients. One lapse in what we know to be our duty in this direction may cost us a desirable patient. Eternal vigilance must be our watchword. Another may say that it would be impossible to give so much attention to these details, and do much else. I reply, that this may be true, but that there is little here that a well trained assistant cannot do, though in many cases it will probably be found necessary to let her know from time to time that not one detail escapes your observation.

Dentistry imposes so many conditions upon those who would follow it successfully that every one should seek to master as many of these as possible. Our success or failure depends in most instances upon ourselves. Success is an exact science, and is won only by faithfulness in little things.

